# Hybrid Service Delivery for voluntary, community and social enterprise organisations working with adults with learning disabilities and/or autism: a realist review protocol

**DOI:** 10.1186/s13643-024-02732-9

**Published:** 2024-12-27

**Authors:** Danielle Varley, Kris Southby, Joanne Trigwell, Sally SJ Brown, Nicola Lines, Amy Hearn, Anne-Marie Bagnall

**Affiliations:** 1 Centre for Health Promotion Research, School of Health, Leeds Beckett University, Leeds, UK; 2 Pyramid, Leeds, UK; 3 100% Digital Leeds, Leeds City Council and NHS (Leeds) West Yorkshire ICB, Leeds, United Kingdom

**Keywords:** Hybrid service delivery, Learning disabilities, Autism, Voluntary and community sector, Digital, Realist review, Health and social care

## Abstract

**Background:**

Delivery of health and care services using a combination of remote and/or in-person channels and digital and/or traditional tools (Hybrid Service Delivery, HSD) is increasingly seen as a way of improving quality and affordability, improving access, personalisation and sustainability, and reducing inequalities. Across the voluntary, community and social enterprise sector (VCSE), using a combination of remote and/or in-person channels and digital and/or traditional tools (HSD) has enabled the essential provision of services for people who have learning disabilities and/or autistic (LDA). However, it is unclear how different tools and channels have been used, what worked well or not well, for whom, and in what circumstances. The aim of this realist review is to explore how VCSE organisations can effectively use digital technologies alongside or instead of in-person activity to provide social care services to adults with learning disabilities and/or autism.

This review protocol is presented in accordance with the Preferred Reporting Items for Systematic Review and Meta-Analysis Protocol (PRISMA-P).

**Methods:**

We will conduct a participatory realist review. Following realist review methodology, and involving people with LDA and organisations who deliver services to them, we will define the scope of the review/theory development, search for and appraise evidence, extract and synthesise findings, and develop the narrative. Using a developed strategy, electronic databases (Academic Search Complete, CINAHL, MEDLINE, PsycInfo, SCOPUS, Social Science Citation Index and Social Policy and Practice) will be searched. A data extraction table will be used to assist in sifting, sorting and organising relevant information from identified studies. For each proposition statement, relevant data from the identified literature will be synthesised and compared with the proposed theory to develop an understanding of how, why and when hybrid delivery works in different settings with different populations.

**Discussion:**

This review aims to collate and synthesise evidence relating to hybrid service delivery in VCSE organisations to provide social care services to LDA adults. By conducting a participatory realist review, we anticipate that the findings will lead to a greater understanding of contextual factors and therefore more relevant recommendations.

**Systematic review registration:**

PROSPERO CRD42024457161.

**Supplementary Information:**

The online version contains supplementary material available at 10.1186/s13643-024-02732-9.

## Background

Delivery of health and care services using a combination of remote and/or in-person channels and digital and/or traditional tools (Hybrid Service Delivery, HSD) is increasingly seen as a way of improving quality and affordability, increasing access, personalisation, and sustainability, and reducing inequalities [1, 2]. ‘Remote delivery’ for ‘adults with intellectual disabilities and/or autism’ is included in care guidance [3] and is increasingly used [4]—a trend accelerated during the COVID-19 pandemic [5, 6].

Across the voluntary, community and social enterprise sectors (VCSE), using a combination of remote and/or in-person channels and digital and/or traditional tools (HSD) has enabled the essential provision of services for people who have learning disabilities and/or are autistic (LDA) [6, 7]. However, it is not clear how different tools and channels have been used, what worked well or not well, for whom, and in what circumstances.

While there is an appetite among some people who have LDA to maintain and extend the use of digital channels and tools for service provision [8, 9], others would prefer to continue with in-person activity [10, 11]. One strand of emerging evidence suggests remote services, for example, should complement, not replace, in-person services [12]. However, more evidence is needed before policy and practice recommendations are made [13, 14]. This includes a better understanding of which service users, under which circumstances, and with what supports can benefit from remote delivery [12]. People who are autistic and/or who have learning disabilities are a heterogeneous group, yet they are often grouped together by health and care services [15] in the absence of an alternative model. VCSE organisations need guidance about how to provide services via remote and digital channels and tools in a personalised way, that takes account of stakeholders’ stated preferences, resources and capabilities.

The increasing role of VCSE organisations and the growth of remote and digital channels and tools for service delivery are both championed by national and local government as processes to achieve more cost-effective, personalised, and accessible services [16]. However, the evidence base about how service delivery using remote and digital channels and tools is used alongside, or instead of, in-person activity (hybrid delivery), now and in the future, is limited and the resultant effect on service delivery unknown. There are concerns that remote and digitally delivered services will proliferate to save money at the expense of quality [17, 18].

This research will fill a specific gap in the evidence base relating to VCSE organisations utilising HSD with people who have LDA. It will contribute to filling a general void in robust evidence relating to the role of the VCSE in delivering social care services and will inform person-centred care by giving VCSE organisations information about how to appropriately deliver services. Now is an opportune moment to consider insights from the pandemic experience and what elements of the “digital turn” might persist in a way that supports inclusion [12, 19].

## Methods/design

### Research aims

The aim of this research is to explore how VCSE organisations can effectively use digital technologies alongside or instead of in-person activity (hybrid delivery) to provide social care services to different adults with learning disabilities and/or autism.

### Research questions

The main questions of this review are the following:

1. How have VCSE organisations carried out hybrid service delivery to adults with learning disabilities and/or autism?
2. What has, and has not, worked well, for whom, and in what circumstances?
3. What are the barriers and enabling factors to hybrid delivery?
4. What does ‘good’ hybrid delivery look like and what should be the criteria for assessing the quality of hybrid VCS delivery?

To achieve the aims and objectives of the project, a *participatory realist review* methodology will be used. This approach involves stakeholders in the review process, drawing on their experiential knowledge to enhance the validity of results and utility of findings, and will lead to a greater understanding of the contextual factors underpinning interventions [20]. However, being guided by input from myriad stakeholders, participatory realist reviews can be less predictable and more time-consuming than traditional systematic reviews. Stakeholders will include an expert advisory group, VCSE workers and VCSE service users with LDA.

The four stages of a realist review [21]—*defining the scope of the review/theory development, searching for and appraising the evidence, extracting and synthesising findings, and developing the narrative*—are used to describe how the review will be conducted.

## Defining the scope of the review/theory development

In a realist review, the phenomenon or intervention being investigated is conceptualised as a theory involving the intervention itself and the context in which it occurs [21]. Prior to searching for evidence, it is therefore necessary to identify outcome-focused programme theories (using a realist framework) and propositions about the intervention and related concepts:

 if ‘X’ happens in ‘Y’ situation then ‘Z’ will happen

These form the basis of frameworks to guide the literature search, data extraction, and synthesis.

The process of theory development in a realist review is inherently participatory. For this review, although the aims and research questions have already been defined during bid writing with academic (KS, AMB, JT) and non-academic (NL, AH) collaborators, two actions will be used to develop preliminary theories concerning hybrid service delivery for adults with learning disabilities and/or autism:

1. *Co-production workshops* with relevant stakeholders (such as service providers and service users) to develop a shared understanding of key issues. Workshops will be online or in-person and audio recorded and transcribed by a member of the project team (SSJB). Workshop data will be analysed to identify the main themes/key issues in relation to the research questions*.* Coding will be inductive and deductive, as necessary. Coding will be conducted by a team member (SSJB) and verified by the project team. Based on the analysis, preliminary theories and accompanying *proposition statements* about VCS organisations’ hybrid service delivery will be produced (e.g. IF video conferencing is used to deliver services THEN it may be more effective for delivering art-based activities to adults with mild-moderate LDA but less effective for services intending to promote social connections or physical activity and with adults with more severe impairments).
2. *Preliminary (purposive) literature searches* to get a ‘feel’ for what is out there and understand the policy background [22]. This will include existing theories (e.g. complete or partial explanations), policy history, and key points of contention with respect to VCS organisations’ hybrid service delivery. Database searches and website searches will be completed (DV). This literature will be analysed using non-systematic data extraction (e.g. identifying the salient points). Findings will be incorporated into preliminary theories.

The preliminary and proposition statements will then be used to guide literature searching and the identification of evidence (e.g. published literature) and be a point of comparison with the identified literature in the final analysis/synthesis. The preliminary theories and proposition statements will be discussed and approved with relevant stakeholders. If the amount of proposition statements produced is unfeasible to carry out literature searches on, the highest priority will be agreed between the research team and relevant stakeholders. This will be decided by holding meetings with relevant stakeholders and experts to hear their perspectives on each statement’s level of importance and relevancy; we will use these perspectives to decide the prioritisation of the statements. Pawson and colleagues [22] caution that completely comprehensive realist reviews may be impossible and recommend that the programme theories to be inspected be agreed upon and prioritised.

## Searching for and appraising evidence

Once the preliminary theory and proposition statements are agreed and/or prioritised, a systematic search will be undertaken by the academic research team to identify evidence to ‘populate’ the proposed theory about VCSE organisations’ hybrid delivery with empirical findings. The proposed theory is a framework for locating, integrating, comparing and contrasting empirical evidence [22].

In comparison to traditional systematic reviews that have one predefined scheme for searching for evidence that aims to be as comprehensive as possible, the search for evidence in a realist review can be iterative and purposive. Realist reviews aim to identify sufficient evidence to answer specific questions or test particular theories, not generate encyclopaedic coverage of all possibly relevant literature [22].

In this review, a ‘main’ search will be carried out using the methods below. We will then carry out individual searches in response to emergent evidence gaps. This will be to try and achieve theoretical saturation across all aspects of the proposed theory [22]. However, this will be bounded by the limited time and financial resources of the project [22].

### Call for evidence (ongoing consultation)

A call for evidence will be issued to local and national civil society organisations on social media, direct email contact, and other professional networks; via academic collaborators on social media and other professional networks. The academic research team will continue to consult with stakeholders at network meetings to report progress and for ideas for additional evidence, as necessary.

### Website searches

Websites of civil society organisations with a focus on intellectual disability, autism, and/or digital inclusion will be hand-searched.

### Electronic database search

A search strategy has been developed and piloted, based on the preliminary selected theories and statements, using the BeHEMoTh framework for guidance [23]. The search strategy is reported in Additional file 1: Table S1.

The following databases will be searched:

- Academic Search Complete
- CINAHL
- MEDLINE
- PsycInfo
- SCOPUS
- Social Science Citation Index
- Social Policy and Practice

Results will be uploaded into review software (Covidence) for management, screening, and data extraction. Two reviewers will screen (i) titles and abstracts, and (ii) full-text articles against the inclusion criteria, with disagreements being resolved by discussion or reference to a third reviewer where needed.

### CLUSTER searching

We expect that quite often single publications will not adequately report all aspects of interventions or programmes. In addition, we expect that studies are not indexed well so can be hard to find in database searches using keywords only. We will use CLUSTER searching [24] to identify publications related to a single intervention. This involves identifying a ‘key pearl citation’ for an intervention and then following up citations, tracing lead authors, identifying unpublished materials, searching Google Scholar, tracking theories, undertaking ancestry searching for early examples and following up on related projects.

### Inclusion and exclusion criteria

Inclusion/exclusion criteria are set out using the PICOS framework (Table 1). These will be reviewed and/or modified following the preliminary theory development.

**Table 1:** Inclusion and exclusion criteria

	Include	Exclude
**P**opulation	• Adults (18 + years) with mild intellectual disabilities • Adults (18 + years) with moderate intellectual disabilities • Adults (18 + years) with severe/profound intellectual disabilities • Adults (18 + years) with moderate autism • Adults (18 + years) with severe/profound autism	• Children (< 18 years)
**I**ntervention	• Services or activities for adults with intellectual disabilities and/or autism delivered online or remotely by voluntary and community sector (VCS) organisations • Services or activities for adults with intellectual disabilities and/or autism delivered in a hybrid format (online/remotely) and in-person) by VCS organisations • Any type of health or social care delivered by VCSE organisations, or services delivered by VCSE organisations that have an overt health and social care impact • Any type of online or remote delivery (e.g. Zoom, telephone)	• Services or activities delivered by statutory organisations (e.g. NHS, local authority, education)
**C**omparison	• In-person delivery • No comparator	
**O**utcomes	• Any individual or organisational outcomes	
**S**tudy design	• Any study design, including conceptual and theoretical papers	
Other	• English language • Data limit for records focused on assessing digital technology published in past 10 years (2014–present)	• Non-English language

## Extracting and synthesising findings

### Data extraction

A data extraction table will be used to assist in sifting, sorting, and organising relevant information from identified studies. While some realist reviews utilise multiple forms for different sources, for this review a single table will be developed. The table will be designed to provide enough flexibility to accommodate the many-sided hypotheses and the multiple sources of evidence that might be included in the review (e.g. different sections may be completed for different sources) [22]. Relevant text from each included study will be copied and/or summarised into the relevant cell of the data extraction table. Two members of the project team will be involved in extracting the data from included articles. Data extraction tables will be reviewed by a third reviewer to ensure accuracy.

The fields of the data extraction table will be aligned with a realist approach—*what works, for whom, and in what context*. Possible fields are below. These fields will be reviewed and/or modified following the preliminary theory development:

- Population (e.g. who is taking part in the intervention? Impairment, age, gender)
- Setting (e.g. location, country)
- Intervention, with reference to the TiDieR checklist [25] (e.g. intervention design, explicit and implicit programme theory^1^; information about delivery; aims and objectives; key factors for delivery/implementation)
- Outcomes (e.g. individual level (service users, family), organisational outcomes (VCSE, commissioners, health and social care), other outcomes)
- Contextual information (e.g. organisational (staff, resources, setting etc.); individual (impairment, support, skills/experience, etc.), other)
- Evidence aligned to individual proposition statements
- Reviewers’ notes and comments

Additional literature searching will be carried out as necessary by the project team to complete any ‘gaps’ in the data extraction table.

### Validity assessment

We will appraise the quality of the information from the relevant records. Due to the complex nature of realist reviews, we will appraise included records by how well they aid the development and testing of theory. Relevance (assessing how well a record addresses the theory of interest) and rigour (assessing the record’s ability to make a methodologically sound contribution to test the theory) checks will be included in the validity assessment. Validity checklists appropriate to the study designs included (for example, Cochrane ROB2 for RCTs [26], ROBINS-I [27] for non-randomised intervention studies, adapted CASP tool for qualitative studies [28], and JBI NOTARI [29] for theoretical papers) will be used to assess rigour. Records will be weighted by their appraisal score during the synthesis; records which do not score highly during the assessment will still be included in the synthesis, but more emphasis will be given to records which score highly, for both relevance and rigour.

### Data synthesis

Data synthesis in a realist review is about refining the proposed theory of how an intervention works [22]. As such, the synthesis process in this review is about refining the proposed theory of hybrid service delivery for adults with learning disabilities and/or autism.

For each proposition statement related to the proposed theory, relevant data from the identified literature will be compared and contrasted with the proposed theory to develop understanding of how, why and when hybrid delivery works in different settings with different populations. Context-Mechanism-Outcome (CMO) chains will be developed for each proposition statement.

## Developing the narrative

A final phase will seek to synthesise the CMO chains for all propositions into a unified theory of hybrid service delivery for adults with learning disabilities and/or autism. The synthesis results will be presented to stakeholders during a deliberative hearing (planned for Summer 2024) to develop reasoned conclusions and recommendations. This will help validate emergent findings and support dissemination (Pawson et al. 2014) (Additional file 2).

## Discussion

This review aims to collate and synthesise evidence relating to the use of digital technologies alongside or instead of in-person activity (hybrid delivery) in VCS organisations to provide social care services to different adults with learning disabilities and/or autism. By conducting a participatory realist review, we anticipate that the findings will lead to a greater understanding of the contextual factors related to the research area. We predict that the findings of the review will be valuable for a wide range of stakeholders involved in the support and care of adults with learning disabilities and/or autism, as well as their families and supporters.

## Supplementary Information

Additional file 1: S1. Search Strategy.

Additional file 2: PRISMA-P Checklist.

## Data Availability

The review’s protocol is available at PROSPERO (CRD42024457161).

## Footnotes

1 It is usually the case in health research that reviewers are challenged to work with studies that have no stated theory. Implicit programme theory can be extracted retrospectively by identifying assumptions about how and why an intervention would work [20]
