# Supplementary material for: Hybrid Service Delivery for voluntary, community and social enterprise organisations working with adults with learning disabilities and/or autism: a realist review protocol
Source: Syst Rev. 2024 Dec 27;13:316. doi: 10.1186/s13643-024-02732-9 (PMC11673684; doi:10.1186/s13643-024-02732-9)
Supplement: Supplementary file 1 — Additional file 1: S1. Search Strategy. [file 13643_2024_2732_MOESM1_ESM.docx]

**Additional file 1: S1. Search Strategy**

1. Autistic Disorder [MeSH]
2. ASC
3. ASD
4. Asperger*
5. autis*
6. autism spectrum disorder*
7. Autism spectrum condition*
8. Child Development Disorder*
9. Kanner* syndrome
10. neurodevelopmental disabilit*
11. Neurodevelopmental disorders [MeSH]
12. Neurodiver*
13. PDD
14. pervasive develop* disorder*
15. 1 OR 2 OR 3 OR 4 OR 5 OR 6 OR 7 OR 8 OR 9 OR 10 OR 11 OR 12 OR 13 OR 14
16. development* disab*
17. Developmental Disabilities [MeSH]
18. Developmental disorder*
19. intellectual development disorder
20. intellectual disability [MeSH]
21. intellectual disorder
22. intellectual impairment
23. learning difficult*
24. learning disab*
25. learning disorders
26. neurocognitive disabilit*
27. specific learning disabilit*
28. Specific Learning Disorder*
29. 16 OR 17 OR 18 OR 19 OR 20 OR 21 OR 22 OR 23 OR 24 OR 25 OR 26 OR 27 OR 28
30. Charity [MeSH]
31. Voluntary sector
32. Voluntary organisation*
33. Third sector
34. Third-sector
35. Community organisation*
36. Community-based
37. Non-project
38. Social enterprise
39. Social sector
40. VCO
41. VCS
42. community enterprise
43. community business
44. Neighbo* organisation
45. community improve*
46. not-for-profit
47. not for profit
48. non-profit
49. non profit
50. co-operative
51. cooperative
52. social entrepreneur*
53. community interest company
54. company limited by guarantee
55. social business
56. social firm
57. affirmative business
58. micro-enterprise*
59. social business
60. community interest corporation
61. social interest company
62. social interest corporation
63. benefit society
64. community anchor
65. community hub
66. mutual aid
67. community AND (manag* OR run OR own* OR control* OR driven OR orient*)
68. 30 OR 31 OR 32 OR 33 OR 34 OR 35 OR 36 OR 37 OR 38 OR 39 OR 40 OR 41 OR 42 OR 43 OR 44 OR 45 OR 46 OR 47 OR 48 OR 49 OR 50 OR 51 OR 52 OR 53 OR 54 OR 55 OR 56 OR 57 OR 58 OR 59 OR 60 OR 61 OR 62 OR 63 OR 64 OR 65 OR 66 OR 67
69. Hybrid
70. Digital
71. Blended
72. Service delivery
73. Face-to-face
74. digital service delivery
75. Technolog*
76. online
77. 69 OR 70 OR 71 OR 72 OR 73 OR 74 OR 75 OR 76
78. 15 OR 29
79. 68 AND 77
80. 78 AND 78

AND

Query 1: Coproduction and empowerment

1. Accessib*
2. attitude*
3. belie*
4. Charrette
5. citizen* jury
6. citizens jury
7. Cocreat*
8. co-creat*
9. codesign
10. co-design
11. Community
12. cooperat*
13. coproduc*
14. co-produc*
15. decision making
16. decision-making
17. design thinking
18. Effective*
19. empower*
20. engage*
21. experienc*
22. explor*
23. feel*
24. governance
25. human-centred design
26. independen*
27. Joint
28. (joint or shared or lay or communit*) AND (decision-making or decision making or policy-making or policy making or service design or planning or governance).
29. Mutual
30. opinion*
31. participatory budget*
32. participatory design
33. perception*
34. perspective*
35. planning
36. policy making
37. policy-making
38. power
39. reflection*
40. self-advocacy
41. self-determination
42. service design
43. Shared
44. Suitab*
45. thought*
46. view*
47. 81 OR 82 OR 83 OR 84 OR 85 OR 86 OR 87 OR 88 OR 89 OR 90 OR 91 OR 92 OR 93 OR 94 OR 95 OR 96 OR 97 OR 98 OR 99 OR 100 OR 101 OR 102 OR 103 OR 104 OR 105 OR 106 OR 107 OR 108 OR 109 OR 110 OR 111 OR 112 OR 113 OR 114 OR 115 OR 116 OR 117 OR 118 OR 119 OR 120 OR 121 OR 122 OR 123 OR 124 OR 125 OR 126
48. 80 AND 127

Query 2: Human rights

1. human right*
2. free will
3. rights
4. accessibility
5. adaptation*
6. determinant*
7. digital equal*
8. digital inequal*
9. digital accessib*
10. digital exclusion
11. digital inclusion
12. digital* divi*
13. digital* inclu*
14. digital* poverty
15. equit*
16. health disparit*
17. health equit*
18. health inequalit*
19. inequalit*
20. social determinant
21. social inequalit*
22. Social justice
23. socioeconomic inequalit*
24. technolog* accept*
25. vulnerable
26. wider determinant
27. 129 OR 130 OR 131 OR 132 OR 133 OR 134 OR 135 OR 136 OR 137 OR 138 OR 139 OR 140 OR 141 OR 142 OR 143 OR 144 OR 145 OR 146 OR 147 OR 148 OR 149 OR 150 OR 151 OR 152 OR 153 OR 154
28. 80 AND 155

Query 3: Infrastructure

1. Infrastructure
2. Network*
3. Transport*
4. Access*
5. Delivery
6. Organisation*
7. Online
8. 157 OR 158 OR 159 OR 160 OR 161 OR 162 OR 163
9. 80 AND 164

Query 4: Safeguarding and harm

1. Attitude* tech*
2. Prevent* tech*
3. Safeguard*
4. victim*
5. (problematic use of the internet)
6. PUI
7. (problematic media use)
8. risk*
9. friendship*
10. anxiety*
11. emotion* AND disturb*
12. negative
13. Harm
14. Protection
15. Exploit*
16. Violen*
17. Maltreat*
18. Abus*
19. Trauma
20. Online harm
21. 166 OR 167 OR 168 OR 169 OR 170 OR 171 OR 172 OR 173 OR 174 OR 175 OR 176 OR 177 OR 178 OR 179 OR 180 OR 181 OR 182 OR 183 OR 184 OR 185
22. 80 AND 186

Query 5: Life skills, knowledge and outcomes

1. Support*
2. Friendship*
3. Cognit* skill*
4. Cognit* improvement*
5. Knowledge
6. Isolation
7. Social*
8. Social interaction*
9. Loneliness
10. Life skill*
11. Outcome*
12. Opportunity*
13. achiev*
14. attain*
15. attention
16. cognit*
17. executive function
18. imitat*
19. joint attent*
20. language*
21. memory
22. perform*
23. reasoning
24. recall
25. social attent*
26. social behav*
27. social orient*
28. 188 OR 189 OR 190 OR 191 OR 192 OR 193 OR 194 OR 195 OR 196 OR 197 OR 198 OR 199 OR 201 OR 201 OR 202 OR 203 OR 204 OR 205 OR 206 OR 207 OR 208 OR 209 OR 210 OR 211 OR 212 OR 213 OR 214
29. 80 AND 215

Query 6: health and wellbeing

1. belonging
2. capabilit*
3. contentment
4. empower*
5. eudaemon*
6. eudaimon*
7. eudemon*
8. flourish*
9. fulfil*
10. happiness
11. health*
12. livability
13. liveability
14. motor skill*
15. overall health
16. physical welfare
17. positive mental health
18. prosper*
19. purpose in life
20. quality of life
21. resilien*
22. salutogen*
23. satisfaction
24. self-esteem
25. sense of belonging
26. sense of community
27. sustainab*
28. thriv*
29. trust*
30. vibran*
31. wellbeing
32. well-being
33. wellness
34. 217 OR 218 OR 219 OR 220 OR 221 OR 222 OR 223 OR 224 OR 225 OR 226 OR 227 OR 228 OR 229 OR 230 OR 231 OR 232 OR 233 OR 234 OR 235 OR 236 OR 237 OR 238 OR 239 OR 240 OR 241 OR 242 OR 243 OR 244 OR 245 OR 246 OR 247 OR 248 OR 249
35. 80 AND 250
